# Evaluation of 5G Positioning Performance Based on UTDoA, AoA and Base-Station Selective Exclusion

**DOI:** 10.3390/s22010101

**Published:** 2021-12-24

**Authors:** Alda Xhafa, José A. del Peral-Rosado, José A. López-Salcedo, Gonzalo Seco-Granados

**Affiliations:** 1 IEEC-CERES, Universitat Autònoma de Barcelona (UAB), 08193 Bellaterra, Barcelona, Spain; jose.salcedo@uab.cat (J.A.L.-S.); gonzalo.seco@uab.cat (G.S.-G.); 2 Airbus Defence and Space, 82024 Taufkirchen, Germany; jose_antonio.del_peral_rosado@airbus.com

**Keywords:** antenna arrays, positioning, 5G cellular networks, new radio, NLoS, integrity, cmWave, hybridization, time of arrival, angle of arrival

## Abstract

Accurate and reliable positioning solution is an important requirement for many applications, for instance, emergency services and vehicular-related use cases. Positioning using cellular signals has emerged as a promising solution in Global Navigation Satellite System (GNSS) challenging environments, such as deep urban canyons. However, harsh working conditions of urban scenarios, such as with dense multipath and Non-Line of Sight (NLoS), remain as one of the key factors causing the detriment of the positioning estimation accuracy. This paper demonstrates that the use of joint Uplink Time Difference of Arrival (UTDoA) and Angle of Arrival (AoA) gives a significant improvement in the position accuracy thanks to the use of antenna arrays. The new advances of this technology enable more accurate user locations by exploiting angular domains of propagation channel in combination with time measurements. Moreover, it is shown that a better localization is achieved by combining the joined UTDoA and AoA with a base-station selective exclusion method that is able to detect and eliminate measurements affected by NLoS. The proposed approach has been tested through simulations based on a deep urban deployment map, which comes with an experimental data file of the user’s position. A sounding reference signal of 5G new radio operating in the centimeter-wave band is used. The obtained results add value to the use of advance antennas in 5G positioning. In addition, they contribute towards the fulfillment of high-accuracy positioning requirements in challenging environments when using cellular networks.

## 1. Introduction

The demand for precise and reliable localization is growing rapidly, being a topic of high interest especially for autonomous and unmanned vehicles [1]. The increasing number of such applications in urban environments requires positioning accuracies at the cm level. Initially, a Global Navigation Satellite System (GNSS) has been the main technology used for positioning purposes as it offers high position and timing accuracy, free access and global coverage. However, GNSS vulnerabilities [2], such as jamming and spoofing, can compromise the reliability and accuracy of the position computation. Moreover, GNSS suffers a severe performance degradation in harsh environments, such as deep urban canyons. Alternative solutions to compensate GNSS limitations and vulnerabilities have been the subject of extensive research, as in [3,4,5]. To this end, fourth-generation (4G) and current fifth-generation (5G) cellular systems have become an aiding source to provide alternative positioning technologies in the absence of GNSS signals in such harsh environments [6].

The Third-Generation Partnership Project (3GPP) has been one of the main motivators setting standard requirements for cellular positioning. The 3GPP 5G New Radio (NR) technology [7] is uniquely positioned to provide added value in terms of enhanced location capabilities. Current localization technologies used to meet these requirements rely on timing-based techniques, i.e., Time of Arrival (ToA), Time Difference of Arrival (TDoA), or Round-Trip Time (RTT) [8], angle-based techniques, i.e., Angle of Arrival (AoA), or their combinations [9,10,11]. All these technologies are expected to play a significant role in achieving accurate user positioning as they possess desirable attributes, namely large bandwidth, massive antenna arrays, centimeter-wave (cmWave) and millimeter-wave (mmWave) transmissions, among others.

However, every category has its own limitations. ToA- and TDoA-based localization methods need an accurate time synchronization, while the AoA method can perform well only when the target is not far away from the sensors. When in harsh working conditions of urban scenarios, these methods working as stand-alone technologies do not achieve the positioning accuracy considered as baseline by the 5G Release 16 [7], i.e., a horizontal positioning error below 10 m for 80% of user equipments (UEs). The most common way to minimize such degradation is the use of hybrid techniques. In this context, the combination of timing-based and angle-based methods is of high interest in order to take advantage of both technologies and provide significant improvement in terms of accuracy. Unfortunately, because there is a predominance of Non-Line-of-Sight (NLoS) conditions in 5G urban transmissions, in the presence of network synchronization error, its achievable positioning capabilities are mainly limited by dense multipath. Some contributions in positioning tend to ignore the presence of such propagation obstacles and consider instead the so-called achievable positioning performance [12]. Some other contributions incorporate the presence of multipath and NLoS, but no mechanisms are implemented to distinguish between Line-of-Sight (LoS)/NLoS situations [13]. In general, most of the algorithms for range-based target localization in NLoS environments [14] require sophisticated mathematical tools, which add computational complexity and therefore increase their execution time.

In this work, we present a realistic deep urban scenario in the presence of NLoS with a tight network synchronization, using uplink signal of 5G NR operating in the cmWave band. The aim of this work is to assess the achievable positioning performance of hybrid Uplink TDoA (UTDoA) and AoA by taking the advantage of using antenna arrays. This technology helps to characterize the effects of 5G cmWave observables in 5G positioning and add value to the use of advance antennas in a relevant application, namely positioning. Moreover, we want to show that, if the positioning computation also considers the detection and the elimination of ranging observations affected by NLoS, the accuracy improves. The proposed approach is applicable to outdoor use cases with high-accuracy positioning requirements. Therefore, this method will pave the way for the fulfillment of high-accuracy positioning requirements in challenging environments when using cellular networks.

The structure of the paper is as follows. Section 2 introduces the signal model, its properties and the observable computation process. Section 3 presents the position algorithm solution and the main integrity-monitoring approaches. Section 4 describes in detail the scenario used in this work and analyses the results. Finally, Section 5 draws  conclusions.

## 2. Signal Model

### 2.1. LTE Positioning Signal Structure

This subsection summarizes the signal models that will be exploited for positioning.

#### 2.1.1. Downlink Signal

In downlink transmission, a dedicated Positioning Reference Signal (PRS) for positioning purposes is specified. It is based on a multi-carrier waveform, which is mainly defined as an Orthogonal Frequency Division Multiplexing (OFDM) scheme. The PRS is specified in 5G standard in TS 38.211 [15]. The received signal is simulated by convolving only one OFDM PRS symbol with a multipath Channel Impulse Response (CIR). The generation of PRS includes two steps: generation of PRS sequences based on Gold sequences and the PRS mapping. The PRS sequences are Quadrature Phase Shift Keying (QPSK) modulated.

#### 2.1.2. Uplink Signal

In uplink transmission, there is not any dedicated pilot for positioning, so the Sounding Reference Signal (SRS) is selected for this purpose. In 5G NR, the SRS generation is implemented according to 3GPP TS 38.211 [15]. The generation of the SRS includes two steps: Zadoff-Chu sequence generation and mapping. Mapping of the signal is done with an interleaving factor of two subcarriers (FR = 2). SRS is transmitted by the UE for uplink channel sounding, which includes the channel estimation (in the frequency domain) and synchronization. The SRS is an uplink OFDM signal filed with a Zadoff–Chu sequence on different subcarriers. It is transmitted as OFDM symbols, which are allocated in specified frequency (subcarrier) and time (slot) positions in 5G NR subframes. Each radio frame consists of 10 subframes and 20 slots. Each slot comprises of seven Single-Carrier Frequency Division Multiple Access (SC-FDMA) symbols with the case of the normal Cyclic Prefix (CP) length configuration. In the frequency domain, resources are grouped in units of 12 subcarriers, occupying a total of 180 kHz with subcarrier spacing of 15 kHz. SRS is transmitted at the last symbol of the slot with full system band area.

### 2.2. Observable Calculation

This subsection describes the process to characterize the observables for each positioning method.

#### 2.2.1. Observable Based on Time of Arrival

The main source of ranging error in cellular networks is due to the effect of multipath, especially with the relative narrow bandwidth. Therefore, our characterization of the observable is based only on the multipath-induced error. The ranging errors are computed for specific propagation conditions, channel models, system bandwidth, signal-to-noise ratio (SNR) levels and time-delay estimator. The LoS conditions are computed according to the distance between receiver and Base Station (BS) and a random probability variable drawn from a uniform distribution between 0 and 1 following the physical-layer abstraction of 5G observables proposed in [16]. The channel models are based on the ones provided in [17]. Since this work is focused on cmWave scenarios, Urban Macro-cell (UMa) and Urban Micro-cell (UMi) scenarios of the 3GPP standard models are considered. Its network operates at frequency bands between 900 MHz and 6 GHz, and the system bandwidth is limited up to 100 MHz. The time-delay estimator computes the maximum peak of the correlation between the received signal and the transmitted positioning pilots within a correlation range. The ranging errors are finally added with the Euclidean distance of user terminal to BS to model the ToA observables formulated as:

(1) $${\hat{\rho }}_{n}=c\cdot {\hat{\tau }}_{n}=\left\|x_{\mathrm{BS},n}-x\right\|+c\cdot \delta t_{5G}+e_{\mathrm{sync},n}+e_{\mathrm{TDE},n},$$

where ${\hat{\tau }}_{n}$ is the time-delay of the 5G signal estimated from the *n*-th cmWave BS (for uplink signals), $x_{\mathrm{BS},n}=\left[x_{\mathrm{BS},n},y_{\mathrm{BS},n},z_{\mathrm{BS},n}\right]$ is the *n*-th BS position, x=[x,y,z] is the receiver position, $\delta t_{5G}$ is the clock offset of the receiver (referenced to 5G time), $e_{\mathrm{sync},n}$ is the BS synchronization error and $e_{\mathrm{TDE},n}$ is the Time-Delay Estimation (TDE) error. The BS synchronization error $e_{\mathrm{sync},n}$ is modeled as in [7], based on a truncated Gaussian distributed random variable with zero mean and standard deviation $\sigma_{\mathrm{sync}}$ within the interval of values $\left[-2\sigma_{\mathrm{sync}},2\sigma_{\mathrm{sync}}\right]$.

Calculating the differences in ToA observables eliminates $\delta t_{5G}$ and yields the TDoA measurement in the time domain. Thus, the *n*-th TDoA observable is computed as the time difference of ranging observables from the serving and neighbor BSs as

(2) $${\hat{\rho }}_{\mathrm{TDoA},n}={\hat{\rho }}_{1}-{\hat{\rho }}_{n+1},$$

for $1\leq n<M_{BS-1}$, where ${\hat{\rho }}_{1}$ is the ranging observable from the serving BS, i.e., the most powerful BS (with highest SNR) at the receiver position, ${\hat{\rho }}_{n+1}$ is the ranging observable from the (*n* + 1)-th neighbor BS and $M_{BS}$ is the total number of BSs used for positioning.

The TDoA estimates are computed with downlink and uplink 5G signals, resulting in the Observed TDoA (OTDoA) and UTDoA location methods, respectively. Since the physical propagation channel is the same for both OTDoA and UTDoA methods, the main performance difference between both methods is due to inter-UE interference. For the downlink positioning, the UE is in charge of measuring the time delays on the reference or pilot signals it receives from serving and neighboring BSs, which actually provide the Euclidean distance between UE and BS. For the case of uplink positioning, the only task on the UE side is to generate and transmit the SRS, which needs less computational effort than calculating a time estimation. This means, in comparison with the downlink part, where the reference signal is generated by the network and the time estimation is done by the UE, the computational effort on the UE is reduced. Both received signals are simulated to estimate the respective ranging errors for different SNR levels. The calculation of SNR differs in the power, gain and noise figure of antennas.

On the other hand, RTT is based on the time-delay estimates on the signals transmitted in the downlink and uplink. The computation process of RTT observables is slightly different from TDoA case. The *n*-th 5G RTT observable is formulated as

(3) $${\hat{\rho }}_{\mathrm{RTT},n}=c\cdot {\hat{\tau }}_{\mathrm{RTT},n}=\left\|x_{\mathrm{BS},n}-x\right\|+e_{\mathrm{RTT},n},$$

where ${\hat{\tau }}_{\mathrm{RTT},n}$ is the two-way time-of-flight of the 5G signal, $x_{\mathrm{BS},n}=\left[x_{\mathrm{BS},n},y_{\mathrm{BS},n},z_{\mathrm{BS},n}\right]$ is the *n*-th BS position, x=[x,y,z] is the receiver position, *c* is the speed of light and $e_{\mathrm{RTT},n}$ is the RTT error, which is defined as $e_{\mathrm{RTT},n}∼N\left(0,{\sigma_{\mathrm{RTT},n}}^{2}\right)$, where ${\sigma_{\mathrm{RTT},n}}^{2}$ is the RTT error variance from the *n*-th BS. This error variance includes the receiver-transmitter synchronization error, noise errors and multipath errors. Note that the position error with RTT follows approximately the same relation with the downlink (DL) and uplink (UL) position error as the corresponding observables do. Moreover, if the noise in UL is bigger than the noise in DL, then the position error with UTDoA should be worse than the position error with the OTDoA. The RTT error is calculated as:

(4) $$e_{\mathrm{RTT},n}=\frac{\left(e_{\mathrm{DL}-\mathrm{TDE},n}+e_{\mathrm{UL}-\mathrm{TDE},n}\right)}{2},$$

therefore

(5) $$\sigma_{\mathrm{RTT},n}=\sqrt{\frac{{\sigma_{\mathrm{DL}-\mathrm{TDE},n}}^{2}+{\sigma_{\mathrm{UL}-\mathrm{TDE},n}}^{2}}{4}},$$

where $\sigma_{\mathrm{RTT},n}$, $\sigma_{\mathrm{DL}-\mathrm{TDE},n}$ and $\sigma_{\mathrm{UL}-\mathrm{TDE},n}$ are the variance of the position error of RTT, OTDoA and UTDoA, respectively. Thanks to the two-way transmission between receiver and transmitter, there is no UE clock offset present in the RTT observables, which relaxes the positioning problem.

#### 2.2.2. Observable Based on Angle of Arrival

**Figure 1:**
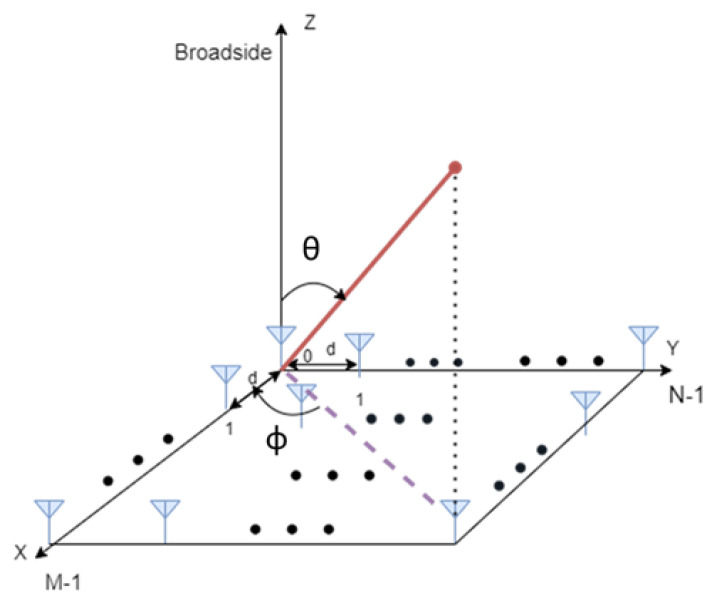
A uniform planar array antenna structure.

The computation process of ranging observables presented in Section 2.2.1 (in terms of physical-layer abstraction) can also be extrapolated to other observables, e.g., angle observable. The computational complexity of the characterization process also scales with the degrees of freedom. For instance, the calculation of angle observables requires an additional dimension for the antenna array orientation with respect to the transmitter, leading to a four-dimensional (4D) interpolator in terms of angle error, antenna array orientation, SNR and propagation probability. In this work, the angular estimation is based on analytical models. Since NLoS measurements are expected to not improve the localization accuracy, this method focuses only on LoS measurements. The Cramér-Rao Bound (CRB) for AoA is used to assess the positioning capabilities in Additive White Gaussian Noise (AWGN) channel, presenting a feasible estimator. The CRB is a well-known lower bound that describes the maximum achievable accuracy of any unbiased estimator in the moderate- to high-SNR region. According to the 5G specifications, the uplink positioning procedure defined in AoA uses the angle of arrival from multiple-array base stations to compute the user position. A Uniform Planar Array (UPA) antenna with *M* antenna elements in the *x*-direction and *N* antenna elements in the *y*-direction, depicted in Figure 1, is used to estimate AoA (composed of the azimuth and elevation angles). The distance between adjacent antenna elements is assigned to be d=λ/2, where $\lambda =c/f_{c}$ is the received signal wavelength, *c* is the speed of light and $f_{c}$ is the carrier frequency.

Sectorized antennas were used, and the orientation of the BS array was chosen so that the array normal vector points approximately in the direction of the target. From Figure 1, one can distinguish the azimuth angle ϕ, which the angle between the *x*-axis and the orthogonal projection of the vector onto the xy-plane, and the elevation angle θ, which is the angle between the vector and its orthogonal projection onto the xy-plane. The boresight direction of the array is aligned with the positive *z*-axis. Knowing the array sectors of each BS sectorized antenna, it is possible to calculate the array direction of BS to UE, which is the angle of incidence at which the signal travels from the UE to the BS.

The angle observable computation process starts first with the calculation of the rotation matrix around *x* axis for each of the orientations of BS arrays using the formula:

(6) $$R_{x}=\left[\begin{array}{ccc} 1 & 0 & 0\\ 0 & \cos\zeta & -\sin\zeta \\ 0 & \sin\zeta & \cos\zeta \end{array}\right]$$

where ζ is the BS array orientation of each of the sectors of the antenna. Then, the calculation of the UE-BS vector’s position $P_{n}^{\prime }$ after rotation is done through the following formula:

(7) $$P_{n}^{\prime }=\left[\begin{array}{c} X^{\prime }\\ Y^{\prime }\\ Z^{\prime } \end{array}\right]=\left[\begin{array}{c} x-x_{BS,n}\\ y-y_{BS,n}\\ z-z_{BS,n} \end{array}\right]R_{x}.$$

Finally,

(8) $$\upsilon_{n,\zeta }=\cos^{-1}\left(\frac{v\cdot P_{n}^{\prime }}{\|v\cdot P_{n}^{\prime }\|}\right)$$

is computed. This is the angle between $P_{n}^{\prime }$ calculated in (7) and $v={\left[0\;0\;1\right]}^{T}$, which is the versor of direction of array (i.e., *z* axis). The chosen array orientation ζ is the one that gives the smallest υ from the results. Once the orientation of the BS arrays is known, it is then possible to calculate the azimuth and elevation angles using the formulas:

(9) $$\phi_{n}=a\tan2\left(\frac{Y^{\prime }}{X^{\prime }}\right),$$

where $Y^{\prime }=\left(y-y_{BS,n}\right)\cos\zeta -\left(z-z_{BS,n}\right)\sin\zeta$ and ζ is the computed array orientation,

(10) $$\theta_{n}=a\cos\left(\frac{Z^{\prime }}{d}\right),$$

where $Z^{\prime }=\left(y-y_{BS,n}\right)\sin\zeta +\left(z-z_{BS,n}\right)\cos\zeta$ and $d=\sqrt{X^{\prime 2}+Y^{\prime 2}+Z^{\prime 2}}$. Both $Y^{\prime }$ and $Z^{\prime }$ are derived from (7).

Since the 5G signal for one symbol is completely known, the formulas of CRB for angular estimation are derived as in [18]:

(11) $$\sigma_{\theta_{n},CRLB}^{2}=\frac{6}{C/\sigma^{2}MNN_{s}{\left(\frac{w_{c}d}{c}\right)}^{2}\cos^{2}\theta_{n}}\frac{1}{{\left[\left(N^{2}-1\right)\sin^{2}\phi_{n}+\left(M^{2}-1\right)\cos^{2}\theta_{n}\right]}^{\prime }}$$

(12) $$\sigma_{\phi_{n},CRLB}^{2}=\frac{6}{C/\sigma^{2}MNN_{s}{\left(\frac{w_{c}d}{c}\right)}^{2}\sin^{2}\theta_{n}}\frac{1}{{\left[\left(N^{2}-1\right)cos^{2}\phi_{n}+\left(M^{2}-1\right)\sin^{2}\theta_{n}\right]}^{\prime }}$$

where $w_{c}=2\pi f_{c}$, $C/\sigma^{2}$ is the SNR of the 5G signal and $N_{s}$ is the number of subcarriers per bandwidth allocated. Note that increasing the number of antenna elements reduces the angle errors. The *n*-th 5G AoA observables of elevation and azimuth are computed using the following formulas, respectively:

(13) $$\theta_{obs,n}=dirBS2UE_{\theta_{n}}+\sigma_{\theta_{k}}\cdot \alpha ,\;\alpha ∼N\left(\mu ,\sigma^{2}\right),$$

(14) $$\phi_{obs,n}=dirBS2UE_{\phi_{n}}+\sigma_{\phi_{k}}\cdot \beta ,\;\beta ∼N\left(\mu ,\sigma^{2}\right),$$

where $\sigma_{\theta_{k}}$ and $\sigma_{\phi_{k}}$ are the angle errors calculated in (11) and (12), $dirBS2UE_{\theta_{n}}$ and $dirBS2UE_{\phi_{n}}$ are the azimuth and elevation angles calculated in (9) and (10), respectively.

## 3. Position Solution and Measurement Exclusion

This section describes the positioning algorithms considered for observables of time of arrival (i.e., UTDoA and RTT) and the one of angle of arrival (AoA). The position computation of the receiver using UTDoA is done with and without the use of NLoS BS exclusion mechanism. The unknown parameter for all cases is the 3D receiver position. These solutions consider both downlink and uplink positioning approaches. Both derivations are applicable in the same way to the location approach.

### 3.1. Location Solution Based on Time of Arrival

Given the known location $x_{BS,n}$ of the *n*-th position among $M_{\mathrm{BS}}$ available BSs, the unknown 3D receiver position x is solved based on the Weighted Least Squares (WLS) classical solution of the UTDoA positioning problem by using the well-known iterative Gauss–Newton (GN) method. The BSs used for positioning are the ones closer in distance with the UE, relative to a reference coordinate.

It is worth noting that ${\hat{\rho }}_{n}$ in (1) is a non-linear function of the user’s position. However, it can be linearized using its Taylor series around a user’s tentative position $\hat{x}=\left[\hat{x},\hat{y},\hat{z}\right]{}^{T}$. Using the tentative position, we calculate the approximate pseudo-range ${\hat{\rho }}_{\mathrm{TDoA},n}$ in (2), for $1\leq n<M_{\mathrm{BS}}-1$. With $M_{\mathrm{BS}}\geq 4$ BSs, it is possible to provide a single solution. The GN solution at the *l*-th iteration is:

(15) $${\hat{x}}_{l}={\hat{x}}_{l-1}+{\left(G_{\mathrm{TDoA},l-1}^{T}W_{\mathrm{TDoA}}^{-1}G_{\mathrm{TDoA},l-1}\right)}^{-1}G_{\mathrm{TDoA},l-1}^{T}W_{\mathrm{TDoA}}^{-1}\left(\rho ({\hat{x}}_{l-1})-\hat{\rho }\right),$$

where

(16) $$\begin{array}{c} \begin{array}{c} \hat{\rho }\left({\hat{x}}_{l-1}\right)={\left[{\hat{\rho }}_{1}\left({\hat{x}}_{l-1}\right),{\hat{\rho }}_{2}\left({\hat{x}}_{l-1}\right),\ldots ,{\hat{\rho }}_{M_{\mathrm{BS}-1}}\left({\hat{x}}_{l-1}\right)\right]}^{T},\\ \rho_{m}\left({\hat{x}}_{l-1}\right)=\|x_{BS,1}-\hat{x}\left\|-\right\|x_{\mathrm{BS},(n+1)}-\hat{x}\|,for\;1\leq n<M_{\mathrm{BS}-1}, \end{array} \end{array}$$

is the vector of measured pseudo-ranges corresponding to the linearization reference position $\hat{x}$,

(17) $$\hat{\rho }={\left[\rho_{\mathrm{TDoA},1},\ldots ,\rho_{\mathrm{TDoA},M_{\mathrm{BS}-1}}\right]}^{T},$$

is the vector of predicted pseudo-ranges corresponding to the real receiver position x, $W_{\mathrm{TDoA}}$ is the weighting matrix formed by the weighting coefficients of the TDoA observables as:

(18) $$W_{\mathrm{TDoA}}=\left[\begin{array}{cccc} \sigma_{1}^{2}+\sigma_{2}^{2} & \sigma_{1}^{2} & \cdots & \sigma_{1}^{2}\\ \sigma_{1}^{2} & \sigma_{1}^{2}+\sigma_{3}^{2} & \cdots & \sigma_{1}^{2}\\ \vdots & \vdots & \ddots & \vdots \\ \sigma_{1}^{2} & \sigma_{1}^{2} & \cdots & \sigma_{1}^{2}+\sigma_{M_{BS}}^{2} \end{array}\right]$$

where $\sigma_{n}^{2}$ is the variance of the *n*-th ranging errors and $G_{\mathrm{TDoA}}$ is the geometry matrix defined as:

(19) $$G_{\mathrm{TDoA},(n,1:3)}=\left[\begin{array}{c} \frac{x_{\mathrm{BS},1}-\hat{x}}{\|x_{\mathrm{BS},1}-\hat{x}\|}-\frac{x_{\mathrm{BS},(n+1)}-\hat{x}}{\|x_{\mathrm{BS},(n+1)}-\hat{x}\|} \end{array}\right].$$

We ensure full rank of $G_{\mathrm{TDoA}}$, which guarantees the invertibility of $G_{\mathrm{TDoA}}^{T}W_{\mathrm{TDoA}}G_{\mathrm{TDoA}}$.

The GN method often converges quickly, especially when the iteration begins with a reference position close enough to the true position. However, if the iteration begins far from the target position, convergence may be slow or may not be achieved at all. These cases are avoided and not taken into account when calculating the position.

The computation position approach is applicable to RTT positioning solution as well, with some minor changes. Using (15),

(20) $$\hat{\rho }\left({\hat{x}}_{l-1}\right)={\left[{\hat{\rho }}_{1}\left({\hat{x}}_{l-1}\right),{\hat{\rho }}_{2}\left({\hat{x}}_{l-1}\right),\ldots ,{\hat{\rho }}_{M_{BS}}\left({\hat{x}}_{l-1}\right)\right]}^{T}$$

is the vector of measured pseudo-ranges corresponding to the linearization reference position $\hat{x}$,

(21) $$\begin{array}{c} \begin{array}{c} \rho_{n}\left({\hat{x}}_{l-1}\right)=\left\|x_{BS,n}-\hat{x}\right\|,for\;1\leq n<M_{BS},\\ \hat{\rho }={\left[\rho_{\mathrm{RTT},1},\cdots ,\rho_{\mathrm{RTT},M_{\mathrm{BS}}}\right]}^{T}, \end{array} \end{array}$$

is the vector of predicted pseudo-ranges corresponding to the real receiver position x, $W_{\mathrm{RTT}}$ is the weighting matrix formed by the weighting coefficients of the RTT observables as:

(22) $$W_{\mathrm{RTT}}=\left[\begin{array}{cccc} \sigma_{1}^{2} & 0 & \cdots & 0\\ 0 & \sigma_{2}^{2} & \cdots & 0\\ \vdots & \vdots & \ddots & \vdots \\ 0 & 0 & \cdots & \sigma_{M_{\mathrm{BS}}}^{2} \end{array}\right]$$

with $\sigma_{n}^{2}$ being the variance of the *n*-th ranging errors and $G_{\mathrm{RTT}}$ is the geometry matrix defined as:

(23) $$G_{\mathrm{RTT},(n,1:3)}=\left[\begin{array}{c} \frac{x_{BS,(m)}-\hat{x}}{\|x_{BS,n}-\hat{x}\|} \end{array}\right],for\;1\leq n<M_{BS}.$$

### 3.2. Location Solution Based on Angle of Arrival

For the case of AoA, the GN solution at the *l*-th iteration is defined as:

(24) $${\hat{x}}_{l}={\hat{x}}_{l-1}+{\left(G_{\mathrm{AoA},(l-1)}^{T}W_{\mathrm{AoA}}^{-1}G_{\mathrm{AoA},(l-1)}\right)}^{-1}G_{\mathrm{AoA},(l-1)}^{T}W_{\mathrm{AoA}}^{-1}b,$$

where $G_{\mathrm{AoA}}$ is the geometry matrix defined as:

(25) $$G_{\mathrm{AoA},(2n,1:3)}=\left[\begin{array}{ccc} \frac{\partial \phi_{n}}{\partial x} & \frac{\partial \phi_{n}}{\partial y} & \frac{\partial \phi_{n}}{\partial z}\\ \frac{\partial \theta_{n}}{\partial x} & \frac{\partial \theta_{n}}{\partial y} & \frac{\partial \theta_{n}}{\partial z} \end{array}\right],$$

composed by the partial derivatives of the azimuth and elevation angles as a function of UE-BS vector’s position coordinates $P_{n}^{\prime }$ calculated in (7). The partial derivatives of both azimuth and elevation angles are derived from the expressions in (9) and (10), respectively. Moreover, $W_{\mathrm{AoA}}$ is the weighting matrix defined as:

(26) $$W_{\mathrm{AoA}}=diag\left(\sigma_{\phi_{n,CRLB}},\sigma_{\theta_{n,CRLB}}\right),$$

and b is the vector of residuals

(27) $$b=\left[\begin{array}{cc} b_{\phi_{n}}; & b_{\theta_{n}} \end{array}\right],$$

being the difference between angle estimation and AoA observables computed in Section 2.2.2. The residuals are wrapped in radians to $\left[\begin{array}{cc} -\pi & \pi \end{array}\right]$.

The location solution derived in (15) can also be used for the hybrid approach, where the total number of BSs used for the hybrid positioning is the sum of the number of the strongest BSs of the TDoA position based-method and those in LoS condition for the angle position based method, i.e., $M=M_{{\mathrm{BS}}_{\mathrm{TDoA}}}+M_{{\mathrm{BS}}_{\mathrm{AoA}}}$. The WLS classical solution of this trilateration problem is similar to the one in (15) and (24), where the geometry matrix $G_{\mathrm{Hyb}}$ is a M×3 matrix defined for $1\leq m\leq M_{BS_{TDoA}}$ as:

(28) $$G_{\mathrm{Hyb},(m,1:3)}=\left[\begin{array}{c} \frac{x_{\mathrm{BS},1}-\hat{x}}{\|x_{\mathrm{BS},1}-\hat{x}\|}-\frac{x_{\mathrm{BS},(m+1)}-\hat{x}}{\|x_{\mathrm{BS},(m+1)}-\hat{x}\|} \end{array}\right].$$

and defined for $M_{BS_{TDoA}}+1\leq m\leq M$ as:

(29) $$G_{\mathrm{Hyb},(m,1:3)}=\left[\begin{array}{ccc} \frac{\partial \phi_{m}}{\partial x} & \frac{\partial \phi_{m}}{\partial y} & \frac{\partial \phi_{m}}{\partial z}\\ \frac{\partial \theta_{m}}{\partial x} & \frac{\partial \theta_{m}}{\partial y} & \frac{\partial \theta_{m}}{\partial z} \end{array}\right],$$

and $W_{\mathrm{Hyb}}$ is the weighting matrix defined as:

(30) $$W_{\mathrm{Hyb}}=blkdiag\left(W_{TDoA},W_{AoA}\right),$$

$W_{TDoA}$, $W_{AoA}$ being the matrix of UTDoA and AoA weighting coefficients, respectively, computed in (18) and (26).

### 3.3. NLoS BS Exlusion Mechanism

As already stated, positioning accuracy is hindered by many propagation effects such as multipath and NLoS, which may appear due to surrounding obstacles, particularly in urban environments. These circumstances have gradually increased the request for more accurate and reliable positioning solution, thus requiring the implementation of alternative measures to minimize such degradation. Our contribution towards this problem consists of a method [19] for detecting faulty measurements from BS affected by NLoS propagation. The method monitors the residuals resulting from the WLS positioning solution, inspired by the approach implemented by Receiver Autonomous Integrity Monitoring (RAIM) techniques in GNSS receivers. The fundamental concept behind the proposed technique is to check the redundancy of range measurements obtained from all available BSs in order to detect one faulty transmitter at a time [20]. The technique includes both a Fault Detection (FD) [21] and a Fault Detection and Exclusion (FDE) functionality (see Algorithm 1).

**Figure 2:**
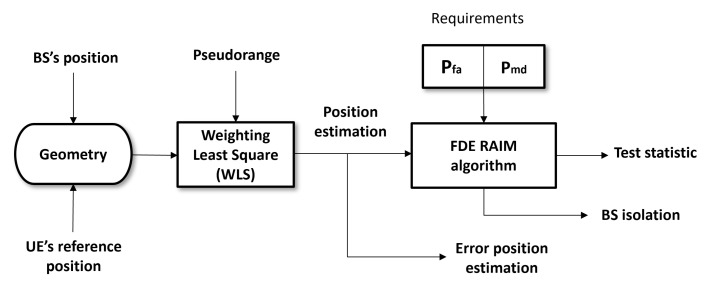
NLOS BS exclusion mechanism.

In conditions of synchronization error among BSs and in the presence of NLoS signals, the proposed method aims to detect large biases induced into the measurements. The workflow diagram of measurement’s monitoring depicted in Figure 2 shows the process of its functionality as follows:

1. Calculate the pseudo-range residuals using all BSs in the scenario. Large residuals indicate that a measurement error (bias) might be present. Generally, to perform a fault detection, there must be at least one redundant observation available. Since we are working with TDoA measurements, a minimum of four BSs are needed to compute a 3D position, five BSs to detect a failure and six BSs to detect and exclude the faulty BS.
2. In order to distinguish between bias-free measurements and those subject to abnormal measurements, a measurable scalar parameter is defined to provide information about pseudo-range measurement errors. This parameter, named test statistic, is related to pseudo-range observations, and it is calculated as the normalized root sum square of the pseudo-range measurement residuals.
3. The test statistic is then compared with a detection threshold *T*. If the test statistic exceeds the given threshold, a bias might be present in the measurements and the faulty BS identification is performed. Otherwise, the solution with all the BSs is used in the scenario.
4. If a failure is detected, we create subsets of BSs by setting one BS as serving for the TDoA measurements and removing one BS from the rest of BSs at a time, so that there will be $\left(M_{{\mathrm{BS}}_{\mathrm{TDoA}}}-1\right)$ subsets, each having $\left(M_{{\mathrm{BS}}_{\mathrm{TDoA}}}-1\right)$ BSs. The detection of the failure is achieved by performing a consistency check through the test statistic parameter for each of the subsets. The subset with the minimum test statistic that does not exceeds the given threshold is chosen to perform the location computation.
5. If the presence of degraded measurement errors is detected, but the faulty BS cannot be identified, the set of BSs that is used for the computation is selected to be the one whose test statistic is the smallest. The candidates’ BSs sets (on which the test statistic is calculated) include the $\left(M_{{\mathrm{BS}}_{\mathrm{TDoA}}}-1\right)$ subsets and also the original set with all the available BS in the scenario. In any case, in such situation when the faulty BS cannot be identified, we recognize that the positioning accuracy remains degraded and does not improve as expected.

#### 3.3.1. Computation of Test Statistic

The method for calculating the test statistic and its theoretical statistical distribution is presented in this subsection. The network provides a WLS estimate of the position based on TDoA measurements. This is done using the linearized measurement equation in (15). The estimated user position $\hat{x}$ and the pseudo-range residual errors $\Delta \rho =\rho \left({\hat{x}}_{l-1}\right)-\hat{\rho }$ obtained from the WLS will later contribute in the computation of the test statistic. The vector of pseudo-range residual errors, Δρ, is the difference between the predicted and the measured pseudo-ranges.

Given the known location $x_{BS,n}$ of the *n*-th position among $M_{BS_{TDoA}}$, available BSs and the estimated user position in (15), it is possible to compute the geometry matrix G as defined in (19). The geometry matrix is decomposed into the signal matrix ($U_{S}$) and noise matrix ($U_{N})$ through QR factorization [22]. The dimension of the noise subspace is ($M_{BS_{TDoA}}-4$). In the absence of any fault, the noise subspace should only contain the noisy contribution of the WLS residuals. and thus it could be modeled as a subspace of zero-mean Gaussian random vectors [23]. However, when a bias is present, the noise subspace will be distorted by the faulty bias and the noise-only condition will not be clear-cut. The QR factorization is preferable instead of performing conventional orthogonal matrix projection because it requires less computation and allows one to easily distinguish the noise from the signal by visualizing the presence of any degradation. These constitutes the building blocks for the detection method.

The residuals on which the detection is implemented are obtained as follows:

(31) $$\varepsilon_{i}=U_{N_{i}}^{T}\;\Delta \rho_{i},$$

where $U_{N}^{T}$ is a $\left(M_{BS_{TDoA}}-4\right)\times \left(M_{BS_{TDoA}}-1\right)$ matrix, whose rows are mutually orthogonal. To normalize the range residuals and remove correlation between data, the whitening process is performed. First, the covariance matrix’s dimensions of the range residuals are made independent by performing principal component analysis. The covariance matrix is calculated as:

(32) $${\mathrm{Cov}}_{res_{i}}=U_{N_{i}}\;{\mathrm{Cov}}_{i}\;U_{N_{i}}^{T},$$

where Cov is the covariance matrix of the pseudo-range errors under the conditions of perfect synchronization error and no bias in the measurements. Then, an eigen decomposition is performed:

(33) $${\mathrm{Cov}}_{res_{i}}=V_{i}\;D_{i}\;V_{i}^{T},$$

where V is an orthogonal rotation matrix composed of eigenvectors of ${\mathrm{Cov}}_{res}$, and D is a diagonal matrix with eigenvalues on the diagonal. $V^{T}$ gives a rotation needed to de-correlate the data. So, we have:

(34) $${\hat{\epsilon }}_{i}={\left(V_{i}\sqrt{D_{i}}\right)}^{-1}\epsilon_{i}.$$

Finally, the test statistic is formed from the Sum of Squared Residuals (SSR):

(35) $${\mathrm{SSR}}_{i}={\hat{\epsilon }}_{i}^{T}{\hat{\epsilon }}_{i},$$

where the *i*-th test statistic for $i=1,2,\cdots \cdots ,M_{BS_{TDoA}}-1$ subsets is given by:

(36) $$T_{i}=\sqrt{{\mathrm{SSR}}_{i}/\left(M_{BS_{TDoA}}-4\right)}.$$

If the measurement errors are normally distributed with zero mean, ${\mathrm{SSR}}_{i}^{2}$ has $\chi^{2}$ distribution with $M_{BS_{TDoA}}-4$ degrees of freedom. The distribution is modified to a non-central $\chi^{2}$ with the same degree of freedom if any measurement error is biased. In this case, the distribution is affected more strongly by geometry through the noncentrality parameter. The decision variable $T_{i}$ is tested against a threshold γ (see next sub-section). Thus, the detection is based on a hypothesis testing where:

$T_{i}\leq \gamma$ : $H_{0}$ is accepted (no biased measurement error),
$T_{i}>\gamma$ : $H_{1}$ is accepted (biased measurement error).

#### 3.3.2. The Selection of Threshold Parameter

The selection of the threshold is done experimentally based on the requirements for false alarm [22]. For a Cumulative Distribution Function (CDF) of test statistic values, the quantile α defines the probability of detecting bias-free BSs subsets. Denoting the threshold γ as that providing a specific probability of false alarm ($P_{\mathrm{fa}}$), the following relationship holds:

(37) $$P_{\mathrm{fa}}\left(\gamma \right)=1-\alpha \left(\gamma \right)=1-\int_{0}^{\gamma }f_{\chi^{2}}\left(T\right)dT.$$

The pseudo-range measurements should be uncorrelated and have a unit variance to follow a desirable normalized $\chi^{2}$ distribution.
**Algorithm 1** NLoS BS detection and exclusion. Procedure from 1 to 20 performs the algorithm with all the available BS, while procedure from 22 to 27 performs the algorithm excluding one BS at a time when the computed test statistic with all the available BS exceeds the given threshold     **Input: $x_{BS,n}$, $\hat{x}$, Cov, γ, $W_{TDoA}$, $\hat{\rho }$, where $n=1,2,\cdots ,M_{BS_{TDoA}}$**     **Output: ${\hat{x}}_{l}$**1:Set NrIter=10 to perform GN solution2:${\hat{x}}_{l}=\hat{x}$3:Set l=04:**while**l≤NrIter**do**5:    l=l+16:    Compute $G_{\mathrm{TDoA},(n,1:3)}=\left[\begin{array}{c} \frac{x_{\mathrm{BS},1}-{\hat{x}}_{l}}{\|x_{\mathrm{BS},1}-{\hat{x}}_{l}\|}-\frac{x_{\mathrm{BS},(n+1)}-{\hat{x}}_{l}}{\|x_{\mathrm{BS},(n+1)}-{\hat{x}}_{l}\|} \end{array}\right]$7:    Compute the pseudo-range error $\rho ({\hat{x}}_{l-1})$.8:    Compute the UE estimated location ${\hat{x}}_{l}$:9:    ${\hat{x}}_{l}={\hat{x}}_{l-1}+{\left(G_{\mathrm{TDoA},l-1}^{T}W_{\mathrm{TDoA}}^{-1}G_{\mathrm{TDoA},l-1}\right)}^{-1}G_{\mathrm{TDoA},l-1}^{T}W_{\mathrm{TDoA}}^{-1}\left(\rho ({\hat{x}}_{l-1})-\hat{\rho }\right)$10:**end while**11:Compute $G_{\mathrm{TDoA}}$ as in 6, where ${\hat{x}}_{l}$ is from 912:Decompose $G_{\mathrm{TDoA}}$ into $U_{S}$ and $U_{N}$ through QR factorization13:Compute the residuals $\varepsilon =U_{N}^{T}\;\Delta \rho$, where $\Delta \rho =(\rho ({\hat{x}}_{l-1})-\hat{\rho })$ from 914:Compute the covariance matrix of the range residual vector ${\mathrm{Cov}}_{res}=U_{N}\;\mathrm{Cov}\;U_{N}^{T}$15:Perform eigen decomposition of ${\mathrm{Cov}}_{res}=V\;D\;V^{T}$16:Whiten the range residual vector as $\hat{\epsilon }={\left(V\sqrt{D}\right)}^{-1}\epsilon$17:Compute the sum of squared residuals: $\mathrm{SSR}={\hat{\epsilon }}^{T}\hat{\epsilon }$18:Compute $T=\sqrt{\mathrm{SSR}/\left(M_{BS_{TDoA}}-4\right)}$19:**if**T≤γ**then**20:    Use the solution ${\hat{x}}_{l}$ in 921:**else**22:    A bias might be present23:    Create subsets of BS by setting the 1st BS as serving and remove one BS from the rest of BS at a time, ${\left\{x_{BS,n^{\prime }}\right\}}_{i}$, where $i\in \{1,2,\cdots ,M_{BS_{TDoA}}-1\}$ and $n^{\prime }\in n-\left\{i+1\right\}$24:    For each of the subsets compute $T_{i}$ following the procedure from 1 to 1825:    Choose ${\left\{x_{BS,n^{\prime }}\right\}}_{i}$ with minimum $T_{i}$ that does not exceeds γ26:    **if** $T_{i}$ found **then**27:        Use solution ${\hat{x}}_{l}$ computed by the corresponding ${\left\{x_{BS,n^{\prime }}\right\}}_{i}$28:    **else**29:        Choose the minimum $T_{i}$ among test statistics computed in 18 and 2430:        Use solution ${\hat{x}}_{l}$ computed by the corresponding group of BSs31:    **end if**32:**end if**

## 4. Simulation Results and Evaluation

In this section, the evaluation of the localization improvements of the uplink-based positioning methods is performed.

### 4.1. Scenario Definition

**Figure 3:**
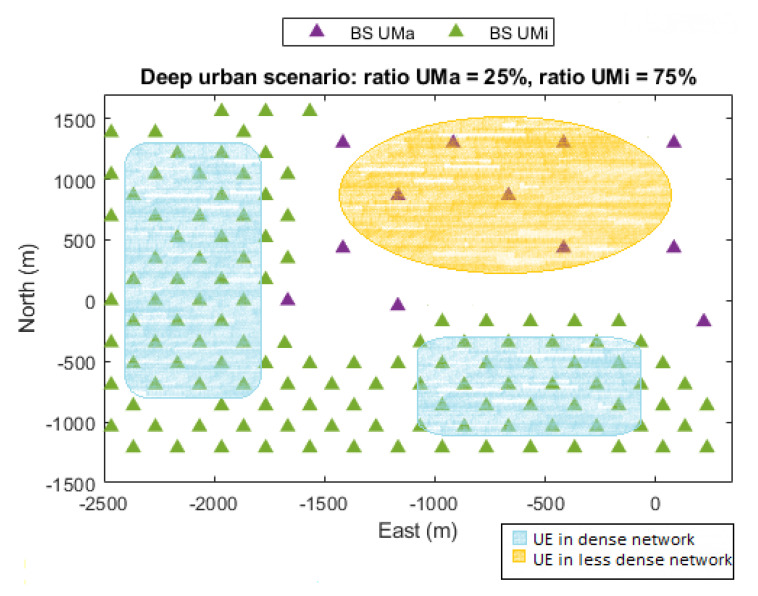
Simulation scenario.

The scenario considered in this work is based on the simulation methodology for outdoor environments within the 5G positioning study in 3GPP Release 16 [7]. It is a predefined Urban Macro-cell (UMa) and Urban Micro-cell (UMi) network scenario based on a deep urban deployment map with BSs deployed above the building rooftops, i.e., BS heights between 20 and 40 m. The deployment consists of a hexagonal grid with seven macro sites, where each has three sectors with an Inter-Site Distance (ISD) around 1732 m in rural areas, 500 m and 200 m in dense urban areas. The method was tested through simulations based on a deep urban deployment map, which comes with an experimental data file of the user’s position. The UEs are randomly placed within the coverage area of the predefined deployment scenario. For reference, see Figure 3. The deep urban area is widely characterized with 75% of the position, while the low density scenario is only shown in 25% of the position. The distance between the BS and UE is used to determine the propagation conditions based on the distance-dependent LoS probability and path-loss models. The multipath channel is stochastically generated following the tabulated channel parameters in 3GPP standard. For each receiver position, the positioning performance is evaluated over 96 iterations of Monte Carlo. The ranging observables are calculated for specific propagation conditions, channel model, system bandwidth (BW), SNR levels and time-delay estimator, and the angle observables are calculated with mathematical models for specific system BW, SNR levels and antenna elements. A 5G PRS and SRS signal with a BW of 20 MHz and 50 MHz at a carrier frequency of 4 GHz, within the cmWave Frequency Range 1 (FR1) of 5G NR and antenna arrays of M=N=11 elements have been considered. The orientation of the BS arrays is set to three typical sectors $\left(0^{\circ },120^{\circ },240^{\circ }\right)$. The network is supposed to have synchronization error among BSs. Table 1 summarizes the simulation parameters used in this work.

### 4.2. Performance Results

To evaluate the 5G positioning performance in dense urban areas, two scenarios have been considered, as mentioned above. Scenario 1 considers a narrow BW of 20 MHz and scenario 2 considers a wide BW of 50 MHz, both at a carrier frequency of 4 GHz with −50 ns or 50 ns Root-Mean-Square (RMS) network synchronization error, distributed randomly every four BS among all available BSs for positioning. Each UE considers a maximum of six BSs to calculate the position. For each of the scenarios, the impact of the angle accuracy of the additional AoA observables and the use of NLoS monitoring method has been analyzed.

The positioning performance is first assessed from a geometric perspective. The additional use of AoA observables with the TDoA observables improves the geometry. This is typically assessed with the Geometric Dilution of Precision (GDOP). Since we are dealing with terrestrial deployment, we are only interested in the horizontal positioning and therefore in the Horizontal Dilution of Precision (HDOP). This parameter for the joint TDoA-AoA is calculated following the derivations in [24], defined as:

(38) $$\mathrm{HDOP}=\sqrt{P_{1,1}^{-1}+P_{2,2}^{-1}},$$

where

(39) $$P=k_{\phi }^{2}G_{\phi }^{T}G_{\phi }+k_{\theta }^{2}G_{\theta }^{T}G_{\theta }+G_{\mathrm{TDoA}}^{T}G_{\mathrm{TDoA}},$$

with $G_{\phi }$, $G_{\theta }$ and $G_{\mathrm{TDoA}}$ being the geometry matrix of elevation angles, azimuth angles and time delays, respectively, computed as in (25) and (19), $k_{\phi }$ and $k_{\theta }$ representing the relationship between variance of time delays with variance of angles (elevation and azimuth, respectively), defined as:

(40) $$k_{\phi }=\frac{\sigma_{\mathrm{TDoA}}}{\sigma_{\phi }},k_{\theta }=\frac{\sigma_{\mathrm{TDoA}}}{\sigma_{\theta }}.$$

These coefficients serve to make the HDOP of AoA dimensionless and comparable to the HDOP of the TDoA. The HDOP value for the angle part can be obtained by extracting the first two terms of the sum in (39) and then using the result in (38). The same method also works for the calculation of HDOP for the time delay part separately. In this case, only the third term of the sum in (39) is needed. Smaller values of HDOP are preferred, meaning that small changes in the measurement will not result in large errors in the location output.

**Figure 4:**
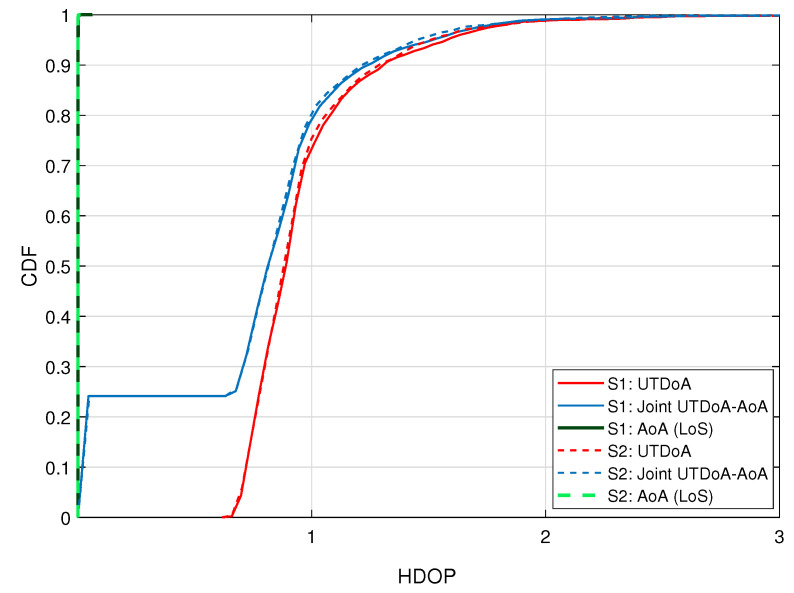
CDF of the HDOP for UTDoA, AoA and hybrid solution. S1 refers to first scenario and S2 refers to the second scenario.

The Cumulative Distribution Function (CDF) of the HDOP is shown in Figure 4 for UTDoA, AoA and the hybrid solution. The HDOP values below 2 are expected to provide precise position estimation. For the cases when AoA and TDoA are treated as a stand-alone positioning method, the HDOP value is independent of the values of $\sigma_{\mathrm{TDoA}}$, $\sigma_{\phi }$ and $\sigma_{\theta }$. In the hybrid approach, their ratio, defined in (40), is a factor that affects the HDOP values. When the values of $k_{\phi }$ and $k_{\theta }$ are very small, the HDOP tends to be equal to the one of TDoA and vice versa. For the case of AoA, the HDOP values are also associated with the size of the deployment area of the positioning system. This is reflected in the small values of the HDOP of AoA, meaning that the BSs are deployed closer in distance.

**Figure 5:**
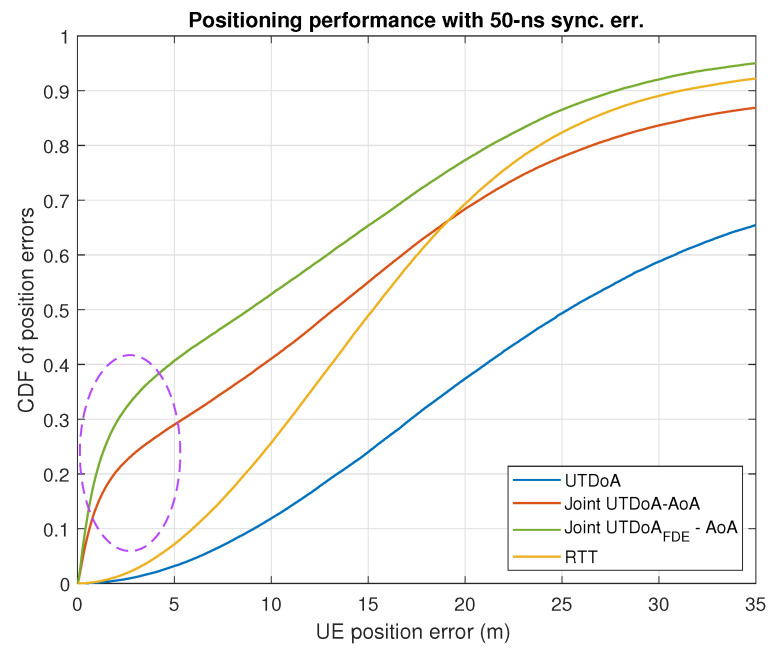
Results for 20 MHz bandwidth with 50 ns sync. err.

**Figure 6:**
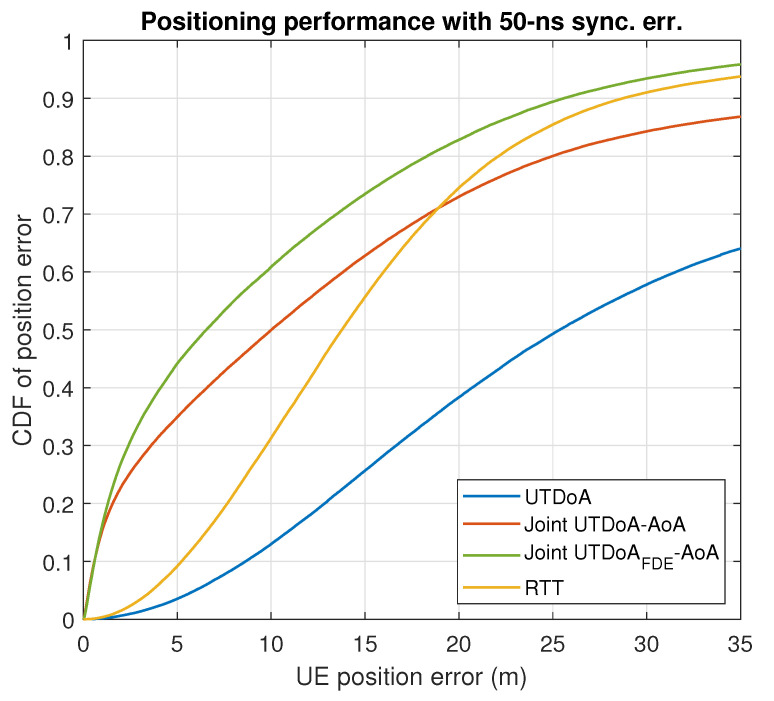
Results for 50 MHz bandwidth with 50 ns sync. err.

**Figure 7:**
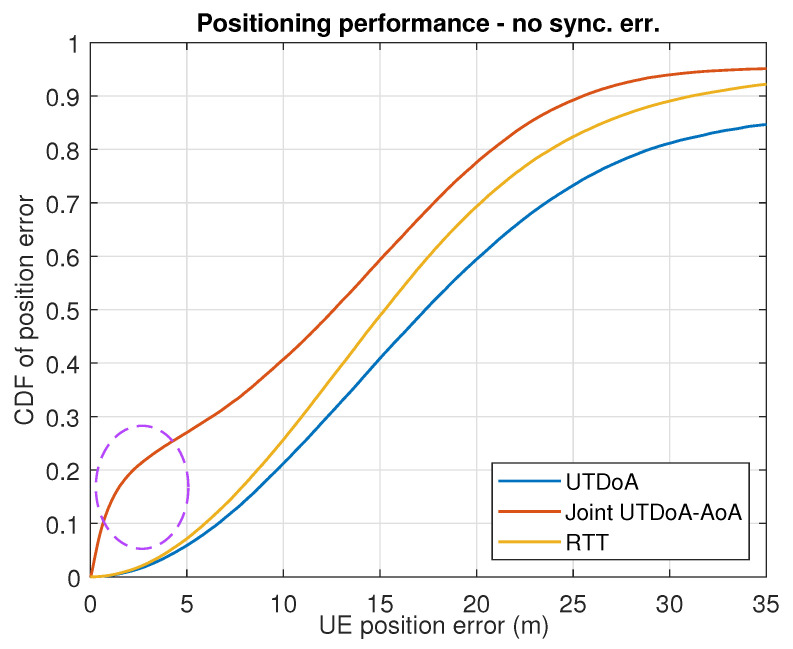
Results for 20 MHz bandwidth with perfect sync.

**Figure 8:**
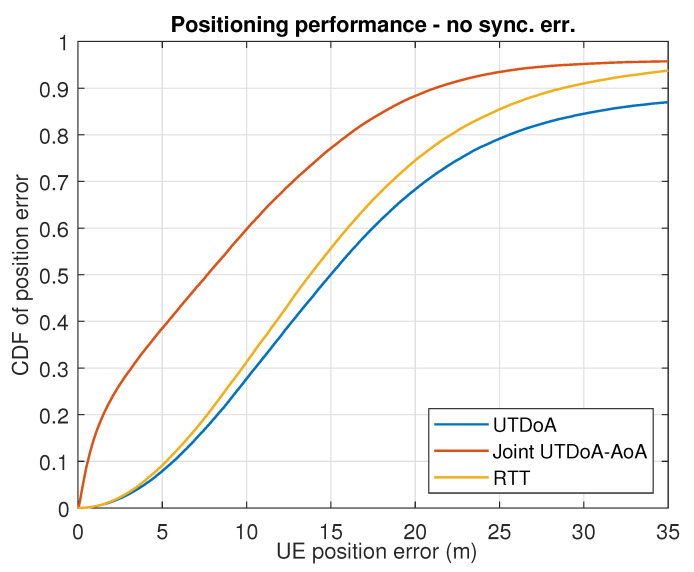
Results for 50 MHz bandwidth with perfect sync.

The positioning accuracy is now evaluated by assessing the actual gain that is obtained in terms of the UE positioning performance. In conditions of synchronization error, with the presence of NLoS and multipath, UTDoA leads to a poor accuracy and reliability of the computation position. This is reflected in the simulation results for both scenarios shown in Figure 5 and Figure 6, where severe degradation errors are clearly seen compared to what is expected in ideal conditions (see Figure 7 and Figure 8). The combination of angle observables with those of time delay has already been shown to provide a significant improvement with UTDoA solutions, where the positioning accuracy is less than 30 m for 80% of the UEs. The maximum horizontal position error is reduced by 27.5%. However, this improvement does not overtake the performance achieved by RTT, which relaxes the constrained positioning problem, i.e., the presence of synchronization error giving a position accuracy improvement of 5%. This can be noticed above the 80th percentile in Figure 5 and Figure 6.

Since there is a predominance of NLoS conditions in 5G urban transmissions, the BS selective solution is of high interest for its ability to eliminate such impairments, in order to achieve a better performance assessment under realistic assumptions while using 5G positioning signals. We therefore analyze the impact of BS exclusion mechanism on the joint UTDoA-AoA positioning performance. The probability of false alarm is set to $P_{\mathrm{fa}}=0.1$, resulting in a threshold of γ=1.4797 m for scenario 1 and γ=1.4879 m for scenario 2, calculated as explained in Section 3.3.2. Results show a significantly improved hybrid solution when the NLoS exclusion mechanism is applied. The maximum horizontal position error is reduced by 40% compared with the results achieved using UTDoA as the stand-alone technology. The use of this mechanism has improved the accuracy of the hybrid method of 18.51%. It also overcomes the performance achieved using RTT and tends to reach the performance of hybrid solution in conditions of perfect synchronization with an improvement of 12.5%. Another observation related to the performance of the positioning methods is the significant improvement in accuracy until the 30% percentile for the first scenario (see dashed circle). This is explained by the presence of more than one LoS angle measurements in the computation solution and ranging measurements in LoS conditions.

Apart from the statistical performance of each method, it is also important to assess the actual gain we obtain through the BS exclusion mechanism in terms of UE positioning performance. The results have shown that it has only met the regulatory requirements for emergency services, i.e., having an accuracy of <50 m on 80% of the cases. However, the use of this method enhances the positioning availability. Table 2 compares the performance of the NLoS monitoring method for both scenarios. From all the BSs that contributed to the positioning computation, 81.86% of them have a synchronization error of −50 ns or 50 ns, and from which 38.75% of the cases, in the group of BSs dedicated for the positioning of each receiver, there is one BS with synchronization error. For 43.11% of the cases, more than one BS has a synchronization error. Only 18.14% of the BSs has perfect synchronization. Having a low $P_{\mathrm{fa}}$, we are expecting that the probability of detecting the faulty BS is high. Results show that for both scenarios, the method was used less than 65% of the time, where in 55% of the cases, the method was able to remove the abnormal measurement. About 7% of the time where the method has been used, the method was not able to remove the faulty BS but managed to remove those whose multipath was large in the majority of cases. The rest of the time, it was not able to perform the monitoring and hence exclude the faulty BS. This is because the method could remove one BS at a time, and therefore, during 43% of the time that there is more than one biased measurement, even if it manages to remove one, the rest of malicious BS will still contribute to the degradation of the accuracy. Finally, the increase in $P_{\mathrm{pfa}}$ increased the percentage of failure of the method, and this will therefore affect in the performance of positioning. For example, when $P_{\mathrm{pfa}}=0.5$, the percentage of the time where the method does not manage to exclude a BS increased almost double compared with the one when $P_{\mathrm{pfa}}=0.1$. All in all, in conditions of a wide bandwidth, the performance is better compared to a narrower bandwidth.

## 5. Conclusions

In this paper, we show the advantages of using array antennas in combination with a time-delay-based positioning method to significantly improve the positioning accuracy in deep urban environments. We performed simulation based on a deep urban deployment map to assess the improvement compare to the case when UTDoA performs as a stand-alone technology. The combination of angle observables with those of time delay has been shown to already provide an improvement of 27.5% in positioning accuracy towards the UTDoA solutions. Additional accuracy can be obtained with the use of a BS exclusion method that we propose. This method allows us to identify and remove abnormal measurements from BS affected by NLoS and synchronization error. The method manages to remove the malicious BS in the majority of cases. The maximum horizontal position error is reduced by 40% compared to UTDoA solution and 18.15% compared to the case without the BS exclusion mechanism. All in all, the obtained results contribute towards the fulfillment of high-accuracy positioning requirements in challenging environments when using cellular networks. Moreover, they show the value of using advance antennas in 5G positioning.

## Figures and Tables

**Table 1:** Simulation parameters.

Parameter	Scenario 1 FR1, 20 MHz	Scenario 2 FR1, 50 MHz
Channel model	Baseline Channel Model based on common assumptions defined related to the channel models of 3GPP TR 38.901	
Carrier frequency	4 GHz	
System Bandwidth	20 MHz	50 MHz
Reference Signal	1-symbol PRS, SRS	
Number of subcarrier	1200	3300
Number of sites	7 (3-sector each)	
Antenna elements	M = N = 11	
Network synchronization assumptions	Perfect sync. and realistic Sync. with T1 = 50 nsec	
Applied positioning algorithm	UTDoA, AoA, RTT joint UTDoA-AoA, joint UTDoA+FDE-AoA, Gauss–Newton algorithm	

**Table 2:** Performance of BS exclusion mechanism in positioning accuracy.

		Scenario 1 (%)	Scenario 2 (%)
**UTDoA measurements**	Sync. err.	81.86	
	No sync. err.	18.14	
**BS for UTDoA positioning**	One BS with sync. err.	38.75	
	More than one BS with sync. err.	43.11	
	No BS with sync. err.	18.14	
**FDE performance**	BS exclusion	55.46	56.47
	BS no exclusion	6.9	6.85

## Data Availability

Not applicable.
